# Targeted therapy for intervertebral disc degeneration: inhibiting apoptosis is a promising treatment strategy

**DOI:** 10.7150/ijms.59171

**Published:** 2021-05-27

**Authors:** Xiao-bo Zhang, Yi-cun Hu, Peng Cheng, Hai-yu Zhou, Xiang-yi Chen, Ding Wu, Rui-hao Zhang, De-chen Yu, Xi-dan Gao, Jin-tao Shi, Kai Zhang, Shao-long Li, Peng-jie Song, Ke-ping Wang

**Affiliations:** 1 Department of Orthopedics, Lanzhou University Second Hospital, Lanzhou, Gansu 730000, PR China.; 2 Department of Orthopedics, Honghui Hospital, Xi'an, Shanxi, 710000, PR China.; 3 Key Laboratory of Bone and Joint Disease Research of Gansu Province, Lanzhou, Gansu 730000, PR China.; 4 Xigu District People's Hospital, Lanzhou, Gansu 730000, PR China.

**Keywords:** intervertebral disc degeneration, death receptor pathway, mitochondrial pathway, endoplasmic reticulum stress pathway, targeted therapy

## Abstract

Intervertebral disc (IVD) degeneration (IDD) is a multifactorial pathological process associated with low back pain (LBP). The pathogenesis is complicated, and the main pathological changes are IVD cell apoptosis and extracellular matrix (ECM) degradation. Apoptotic cell loss leads to ECM degradation, which plays an essential role in IDD pathogenesis. Apoptosis regulation may be a potential attractive therapeutic strategy for IDD. Previous studies have shown that IVD cell apoptosis is mainly induced by the death receptor pathway, mitochondrial pathway, and endoplasmic reticulum stress (ERS) pathway. This article mainly summarizes the factors that induce IDD and apoptosis, the relationship between the three apoptotic pathways and IDD, and potential therapeutic strategies. Preliminary animal and cell experiments show that targeting apoptotic pathway genes or drug inhibition can effectively inhibit IVD cell apoptosis and slow IDD progression. Targeted apoptotic pathway inhibition may be an effective strategy to alleviate IDD at the gene level. This manuscript provides new insights and ideas for IDD therapy.

## Introduction

As the most common cause of chronic low back pain (LBP), IDD affects 80% of the population worldwide, and the prevalence in adults over 60 years old is as high as 68% 1, 2. Chronic LBP has become a severe medical and social problem globally and is one of the causes of complete disability in middle-aged and older adults 3. Intervertebral disc (IVD) degeneration (IDD) results in pathological and anatomical changes in spinal structure to a large extent, which increases the risk of LBP. However, clinical treatment still targets symptoms (including conservative and surgical methods), which cannot limit disease progression or cure the disease at the etiological level. Due to the difficulty of long-term adherence to strict bed rest and damage to the body due to long-term painkiller use, most patients eventually choose surgical treatment. However, surgery still has risks of high invasiveness and recurrence and may even further exacerbate the existing injury. The high cost of surgery has brought substantial economic burdens to families and society. Consequently, a new treatment type is urgently needed to change the current treatment model.

In recent years, targeted therapy has been widely studied. This treatment strategy can significantly reduce surgical risks and postoperative complications 4, 5. As the important pathological change in IDD, apoptosis has broad application prospects in targeted therapy. The death receptor, mitochondria, and endoplasmic reticulum stress (ERS) pathways, as which are classic apoptotic pathways, are involved in IDD occurrence and development and even in IVD cell apoptosis after trauma 6. Apoptosis induces IDD occurrence and worsening and plays roles in different grades of disease. Compared with those in moderate and severe IDD, the death receptor and ERS pathways play more critical roles in mild IDD. The mitochondrial pathway plays a more crucial role in moderate and severe IDD than in mild IDD 7. The activation of these three classic apoptotic pathways is an essential factor leading to worsened IDD. Apoptotic pathway inhibition by technical means may be an effective targeted therapy.

## IDD Etiology

Although some progress has been made in biological research, the IVD microenvironment remains challenging in the context of natural therapy development. Normal IVDs are located in low oxygen, low nutrition, high osmotic pressure, and high mechanical strength environments 8. Microenvironmental change in the IVD is a crucial reason for IDD. In addition, given the development of genetic and proteomics technology in recent decades, the understanding of the origin of IDD has evolved from the classic environmental impact theory to a complex disease with multiple etiologies, whose molecular and genetic basis is associated with many risk factors. Although the current research cannot fully explain the etiology and pathogenesis of IDD, it has been confirmed that IDD is associated with biomechanical factors, inflammatory factors, changes in enzyme activity, apoptosis, and susceptibility genes 9. When IDD occurs, IVDs show morphological changes such as decreased proteoglycan levels, reduced water content in the nucleus pulposus (NP), NP cell (NPC) death, and ECM decomposition.

Mechanical load is one of the critical factors in IDD, forming a vicious cycle with IVD biological changes 10. IDD is characterized by increased proinflammatory cytokines, such as tumor necrosis factor-alpha (TNF-α), interleukin (IL), and matrix metalloproteinase (MMP), which promote ECM degradation and phenotypic changes in IVD cells 11, 12. When the cellular anabolic response is unbalanced, IDD, IVD protrusion, and nerve root pain occur 13. Chemokines released from degenerated IVDs promote immune cell infiltration and activation, further amplifying the inflammatory cascade.

Apoptosis theory has been a research hotspot in recent years. Death of IVD cells, such as annulus fibrosus cells (AFCs), NPCs, or cartilaginous endplate (CEP) cells, caused by activation of the apoptosis pathway is a fundamental cause of IDD that leads to IVD cell loss and ECM degradation and further accelerates disease progression 14. Elucidation of molecular changes in IDD can help clinicians inform patient lifestyle choices, identify beneficial or harmful supplements, or provide new biological, genetic, or stem cell therapies.

## Apoptosis

Apoptosis is different from programmed cell death (PCD) because cell death can occur during physiological development in the absence of apoptotic characteristics 15. Apoptosis can be roughly divided into the following stages: an apoptotic signal is received, which activates the proteolytic enzyme by regulating the interaction between molecules and stimulates the process. IDD-associated apoptosis pathways include the exogenous death receptor pathway, endogenous mitochondrial pathway, and ERS pathway. These pathways induce IDD by mediating IVD cell death, which is the leading cause of IDD induced by apoptotic factors 16, 17.

### Death receptor pathway

**Figure 1:**
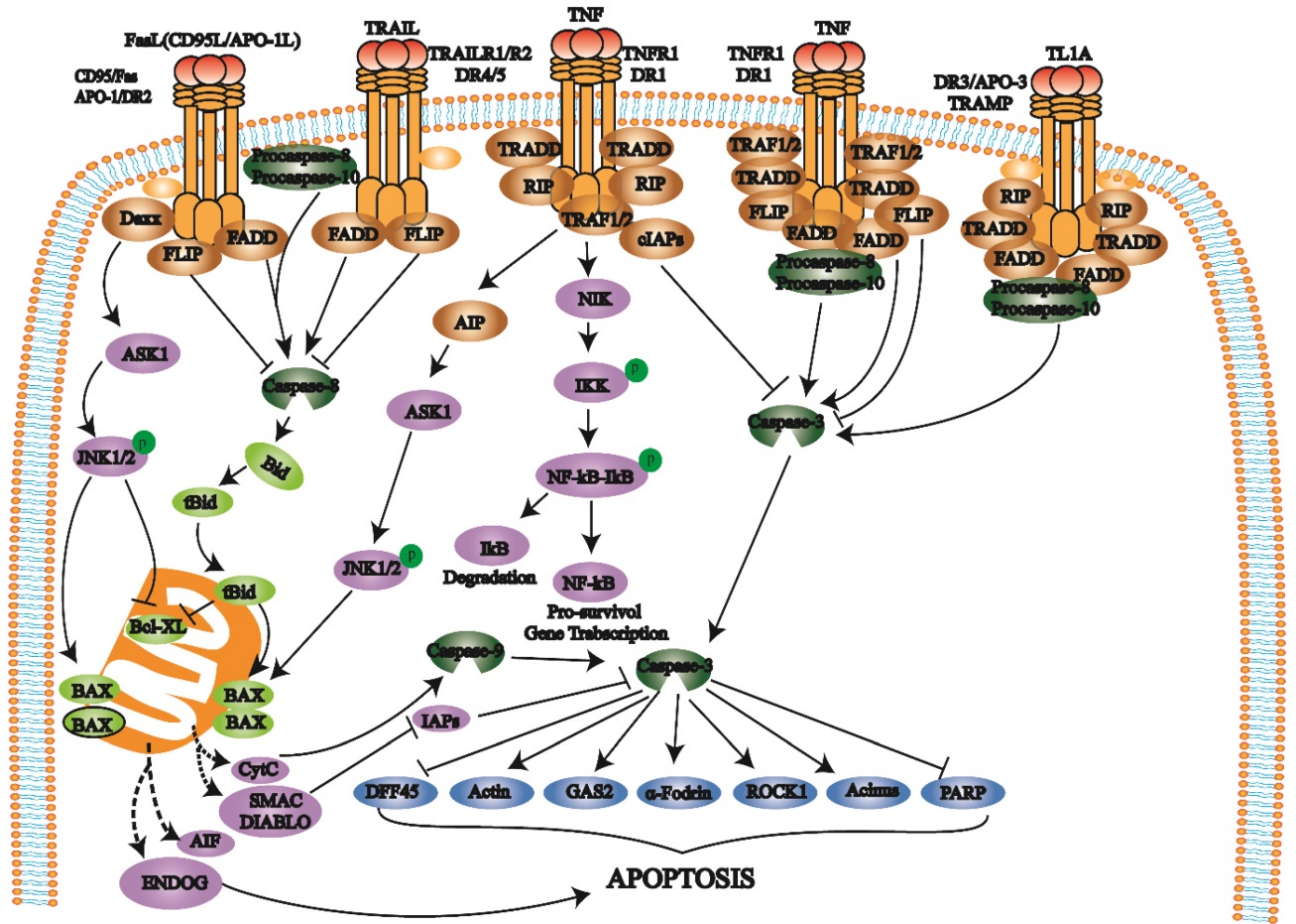
Death receptors (DRs) belong to the TNFR superfamily and share a Cys-rich extracellular domain and intracellular death domain (DD). At present, the known death receptor-ligand pairs mainly include Fas (Apo-1, CD95) - FasL (CD95L), TNFR1 (DR1) - TNF, TRAILR1 (DR4) - TRAIL (APO-2L), TRAILR2 (DR5) - TRAIL (APO-2L), and DR3 (APO-3, TRAMP) - TL1A. When death receptors bind to specific death ligands, they receive extracellular death signals and activate the intracellular apoptosis mechanism. Caspase-8 induces apoptosis by activating the effector protein caspase-3.

The death receptor pathway involves various external factors that promote and mediate apoptosis through different death receptor signaling pathways. The death receptor pathway is a type of exogenous apoptosis 18, a critical way to regulate apoptosis and is crucial for cell proliferation and apoptosis balance 17, 18. There are three death receptor pathways: Fas, tumor necrosis factor receptor 1 (TNFR1), and TNF-related apoptosis-inducing ligand (TRAIL) 19 (Fig. 1).

The Fas pathway occurs through FasL-induced Fas trimer formation. The Fas trimer recruits and binds to the N-terminal death domain and Fas-associated protein with the death domain (FADD) of cytoplasmic adaptor proteins, which transmits apoptotic signals to procaspase-8. Fas-FasL-FADD-procaspase-8 forms a death-inducing signaling complex (DISC) 20. Procaspase-8 then cleaves itself to form active caspase-8 21. Subsequently, caspase-8 activates the effector protein caspase-3, which eventually leads to protease hydrolysis and a series of enzyme-linked reactions that cause DNA degradation and cell death. The TNF trimer binding to TNFR1 induces DD aggregation to recruit the adaptor protein TRADD, which then recruits TRAF2, RIP, and FADD. TRAF2 and RIP activate the NF-κB and JNK/AP signaling pathways, while FADD activates the caspase cascade. FADD recruits and activates procaspase-8 through the DED and then induces the caspase cascade to mediate apoptosis; FADD also recruits FLIP and inhibits the active release of caspase-8. Additionally, TRAF-2 can activate NF-κB-induced kinase (NIK) and IκB kinase (IKK) through phosphorylation. IKK phosphorylates IκB, releases NF-κB, activates antiapoptotic gene expression and promotes cell survival. After TRAILR1 and TRAILR2 bind to the ligand TRAIL, they combine with FADD through the DD to recruit procaspase-8 to form DISC and activate caspase-3, thereby mediating apoptosis.

### Mitochondrial apoptosis pathway

**Figure 2:**
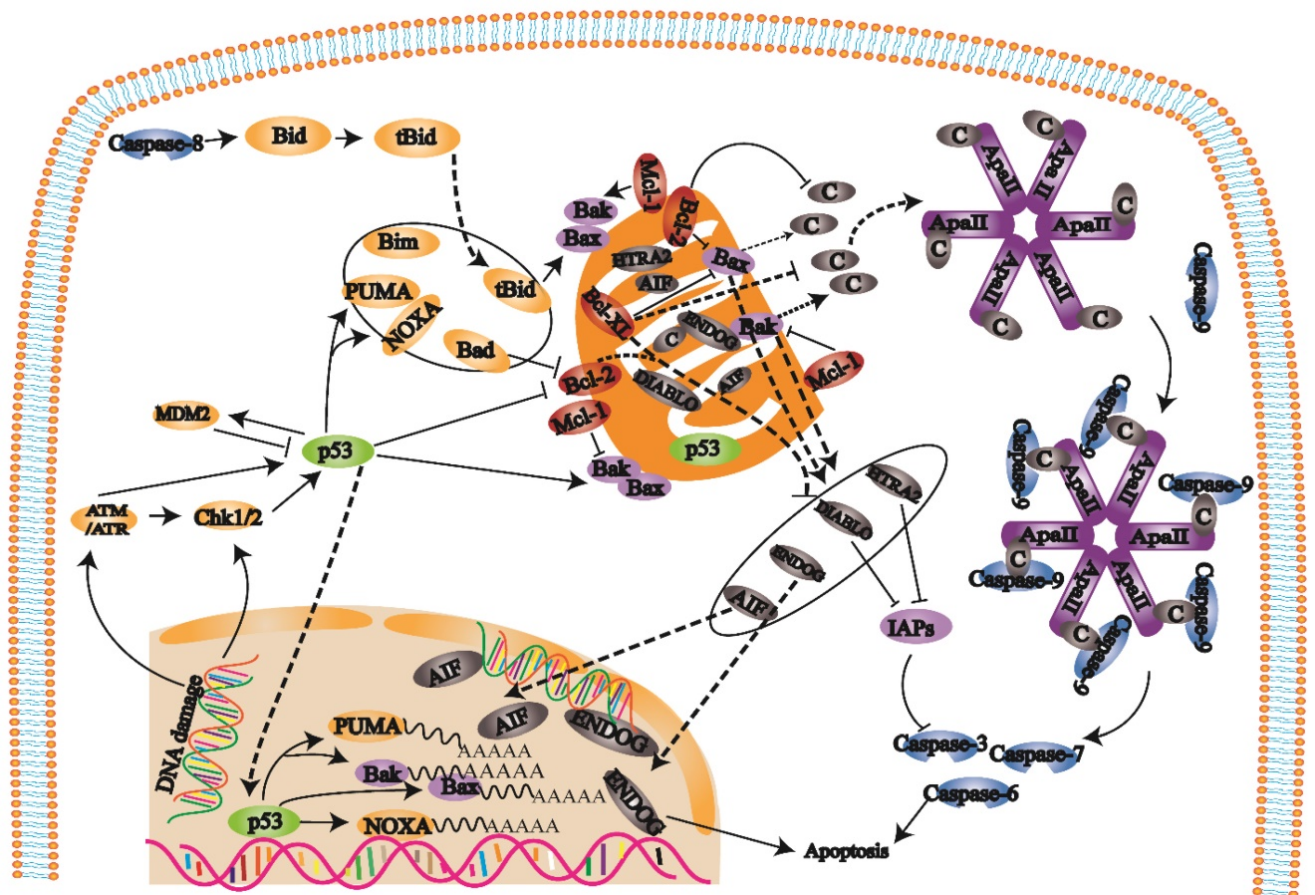
When cells are exposed to internal apoptosis-stimulating factors such as oncogene activation, DNA damage, hypoxia, loss of growth factors, and activation of the internal mitochondrial apoptosis pathway, caspase-8 triggers apoptosis. In this pathway, Bcl-2 family proteins control mitochondrial outer membrane permeability by regulating membrane potential. When the mitochondrial membrane potential is decreased, mitochondrial membrane permeability is increased, and the proapoptotic factors in mitochondria are released into the cytoplasm, which eventually induces apoptosis and activates caspase-3.

B-cell lymphoma-2 (Bcl-2) family proteins trigger the internal mitochondrial pathway by controlling mitochondrial outer membrane permeability through the synergistic effects of proapoptotic and antiapoptotic members 22, 23 (Fig. 2). When the mitochondrial membrane potential is decreased, membrane permeability increases, and proapoptotic factors are released into the cytoplasm. After cytochrome c is released into the cell, the apoptotic complex is formed with ATP and dATP assistance. The apoptotic complex recruits and activates procaspase-9 to form the caspase-9 holoenzyme. Caspase-9 further activates the effectors caspase-3 and caspase-7, which initiates the caspase cascade and ultimately leads to apoptosis 24. Inhibitors of apoptosis proteins (IAPs) can inhibit caspase-3 and caspase-7 activation. Second mitochondria-derived activator of caspase/direct inhibitor of apoptosis-binding protein with low pl (Smac/DIABLO) and HTRA2/OMI are released from mitochondria and bind to IAPs to hinder the effect of IAPs and indirectly promote apoptosis. Changes in mitochondrial membrane potential result in the release of AIF and ENDOG into the cell. These factors transport to the nucleus cause chromosome condensation and DNA fragmentation to induce apoptosis 25, 26.

### ERS pathway

The ER regulates protein synthesis, folding and aggregation after synthesis and maintains the cell stress response and cell calcium levels. An imbalance in Ca^2+^ ions in the ER cavity and an increase in misfolded or unfolded proteins cause ERS 27. ERS reduces protein synthesis, increases protein folding, and maintains Ca^2+^ homeostasis in cells, but an excessive stress response can trigger and promote apoptosis 28.

When cells develop a high-intensity unfolded protein response (UPR), three transmembrane protein kinase ribonucleic acid (RNA)-like endoplasmic reticulum kinases (PERKs), inositol-requiring enzyme 1 (IRE1), and activating transcription factor 6 (ATF6) on the ER play repair roles. Furthermore, these proteins also initiate three apoptotic pathways mediated by ERS 29.

**Figure 3:**
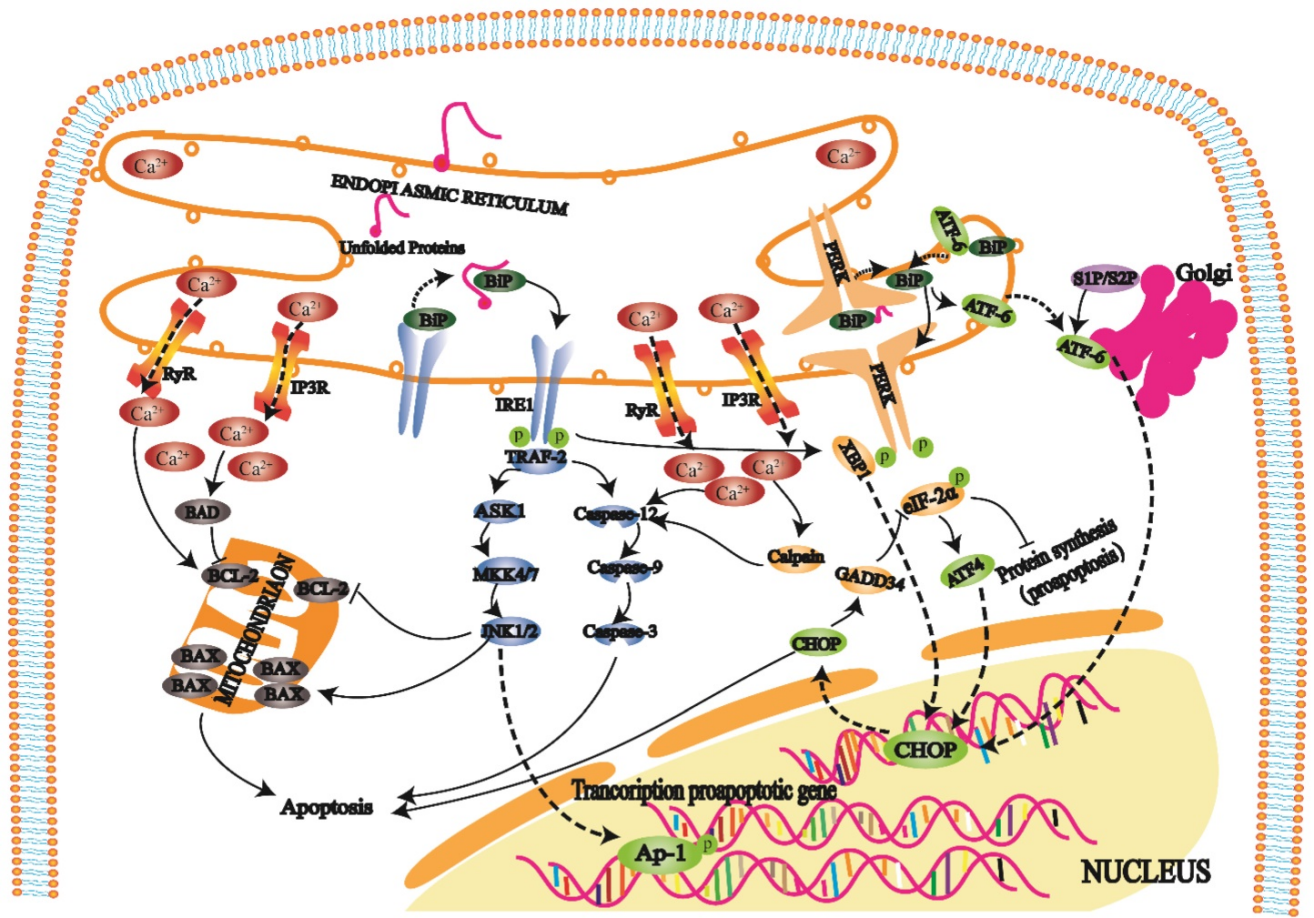
The UPR is an important self-protection mechanism of cells against ERS. When long-term or high-intensity UPR occurs in cells, three transmembrane proteins, PERK, IRE1, and ATF6, on the ER can play repair roles and initiate three apoptosis pathways mediated by ERS. On the one hand, an imbalance in Ca^2+^ affects mitochondrial and Bcl-2 family protein activities, which leads to apoptosis. On the other hand, Ca^2+^ imbalance activates intracellular calpain, affecting apoptosis by activating the caspase cascade.

PERK is a protein kinase distributed on the endoplasmic reticulum membrane (Fig. 3). When this protein is folded normally, it binds with molecular chaperones such as BIP/GRP78 to form a stable complex; when a protein is not folded normally, the incorrectly folded protein binds to BiP/GRP78 and competitively interferes with the interaction between BiP/GRP78 and PERK. The released PERK is activated by oligomerization and undergoes reverse autophosphorylation. Activated PERK can phosphorylate the α subunit of eukaryotic translation initiation factor 2α (eIF-2α). In the early stage of the stress response, phosphorylated eIF-2α inhibits protein translation and synthesis, reduces protein folding in the ER, and has a protective effect on cells. With increasing stress response time and intensity, phosphorylated eIF-2α induces transcription factor activating transcription factor 4 (ATF4), promoting expression of the apoptotic signal molecule C/EBP-homologous protein (CHOP)/GADD153 and causing apoptosis 30, 31.

IRE1 is another protein kinase distributed on the endoplasmic reticulum membrane (Fig. 3). When unfolded proteins accumulate in ER, the IRE1-BiP/GRP78 complex dissociates, and released IRE1 is activated. During apoptosis, activated IRE1 recruits the cytoplasmic regulatory protein TRAF-2 and indirectly recruits and activates c-Jun N-terminal kinase. Subsequently, c-Jun N-terminal kinase can inhibit Bcl-2 family protein activity by phosphorylation and promote apoptosis. On the other hand, activated TRAF-2 activates caspase-12 and initiates the caspase cascade. IRE1 has ribonuclease activity, cleaves X box binding protein 1 (XBP1) messenger RNA (mRNA), promotes XBP1 mRNA maturation, enhances chaperone protein and CHO transcription, and leads to apoptosis 32, 33.

ATF6 is a transmembrane protein on the ER membrane (Fig. 3). The N-terminal intracellular region of ATF6 contains a DNA transcriptional activation domain and B-ZIP nuclear localization signal. In the nonstress state, ATF6 is distributed on the ER membrane in the form of a zymogen. During ERS, ATF6 is transferred to the Golgi body in vesicles. ATF6 is activated by S1P and S2P cleavage in the Golgi apparatus and then migrates to the nucleus via nuclear localization signals. The transcription of ERS genes, including CHOP/GADD153, is induced in the nucleus 34, 35.

Under physiological conditions, the ER mainly releases Ca^2+^ into the cytoplasm through RyR and IP3R channels and pumps Ca^2+^ into the ER cavity through calcium pumps to maintain Ca^2+^ homeostasis (Fig. 3). When the ER receives stress signals, Ca^2+^ homeostasis is broken, and a large amount of Ca^2+^ enters the cell and mitochondria. Ca^2+^ influx affects mitochondrial and Bcl-2 family protein activity and induces cell apoptosis. In addition, it can also activate intracellular calpain, affecting apoptosis by activating the caspase cascade.

## The Death receptor pathway and IDD

### The Fas pathway induces IDD

When the external environment changes or some internal factors activate the death receptor pathway, IVD cell death can lead to IDD pathogenesis. The current research on IDD mainly focuses on the Fas pathway, and this paper mainly describes Fas pathway research progress in IDD.

sFas and sFasL may play a role in IDD. The Fas mRNA level in degenerated NPCs was significantly higher than that in normal cells 36. In contrast, FasL showed the opposite pattern, and the expression levels in degenerative and typical environments were different 36. Because the NP is highly hydrated and has a low cell density, direct cell contact is not common, and cytokine paracrine signaling may play an important role. In this context, sFas and sFasL may be the functional executors of their membrane forms 36. In addition to promoting NPC apoptosis, the death receptor pathway can also promote AFC apoptosis, leading to IDD occurrence 37. Serum deprivation (0%) can activate caspase-8 and caspase-3 in AFCs 38. However, the loss of mitochondrial membrane potential and cytochrome c release to the cytoplasm were not found in serum-deprivation conditions, indicating that AFC apoptosis after serum deprivation was mediated by the Fas pathway and did not involve mitochondria 38. The Fas pathway can also act on the CEP. In degenerative IVDs, FasL expression and the degree of apoptosis in CEP cells are higher than those in undifferentiated IVD cells. Studies have shown that FasL expression and the percentage of apoptotic CEP cells are significantly associated with patient age 39.

In recent years, the role of FasL in IDD has been controversial. FasL not only protects against IDD but also has different effects on different aspects of the disease. Han et al. 40 noted that FasL may play dual roles in regulating NPC apoptosis. FasL can induce immune cell apoptosis and mediate inflammation 41.

### Fas/FasL gene polymorphisms

Genetic analysis and genome-wide research and technology show that genes are associated with the progress and development of conditions. In different populations, IDD is associated with different genes 42. Fas/FasL is the most widely studied apoptotic pathway, and gene polymorphisms are involved in the occurrence and severity of IDD. Zhu et al. 43 found that the Fas 1377 G/A and FasL 844 C/T polymorphisms were associated with IDD severity in the Chinese Han population. He and colleagues hypothesized that these two polymorphisms could be used as independent genetic markers to predict IDD occurrence. Compared with those with the CC genotype, patients with the TT genotype had higher risk of LDH; therefore, FasL-844 TT may be associated with more severe IDD. Furthermore, a significant association between FasL-844 T/C and waist burden, smoking, and age was found 36.

As previously described, the role of FasL in IDD is controversial. Since FasL expression in IDD is reduced, most scholars believe that FasL expression by NPCs has a protective effect on IVDs 44. Huang et al. 45 reported that the Fas G allele (rs1800682) increased the risk of IDD, and the FasL T allele (rs763110) had the opposite effect. However, there are different views: FasL is harmful to IVDs 46. Han et al. 47 found that FasL could promote NPC apoptosis by upregulating Fas expression in NPCs, and apoptosis was dose-dependent regarding FasL (Table 1).

Therefore, the role of FasL has not been fully elucidated. It is necessary to continue to study the relationship between FasL and IDD and the specific mechanism. In addition, specific IDD phenotypes are associated with particular genotypes, single nucleotide polymorphisms, and gene-gene and gene-environment interactions 42. Although studies have verified this correlation, the extent of genetic and environmental factor influences is still unknown. There is an opportunity to develop new types of diagnosis, prevention, and treatment for this disease.

### Inflammatory factors

Inflammation is an essential factor leading to IDD. The known inflammatory factors associated with IDD include TNF-α, IL-1, and IL-2 48. In the NP tissues of patients with LDH, the IL-2 expression level is upregulated. IL-2 inhibits human NPCs (HNPCs) proliferation in a dose-dependent manner, activating the death receptor pathway to induce apoptosis and ECM degradation 49. Various studies have shown that FasL is an essential factor in the production of proinflammatory cytokines, which is consistent with the *in vitro* study by Yoshida et al. on rabbit IVDs 50, 51 (Table 2). FasL is also a vital regulator that induces invasive external cell apoptosis 52. Although inflammatory mediators, especially TNF-α and IL-1, induce IVD cell apoptosis, the experimental technology is relatively mature. The exact mechanism of action between inflammatory mediators and Fas signaling remains unclear. Due to the key role of the Fas pathway in promoting inflammation, we hypothesize that Fas and IDD inflammatory factors may have synergistic effects. Fas signaling inhibition may partially inhibit the inflammatory response of IVDs.

### Participation in immune mechanisms

The blood-NP barrier is composed of the AF, the CEP, and immune molecules. Under normal conditions, the NP is isolated from the host immune system. When the AF ruptures or other factors cause NP tissue to be exposed to the immune system, the barrier is damaged, and T and B cells will be activated to cause an autoimmune response. The immune response associated with the NP involves various downstream proteins plays a vital role in the entire IDD process 53. The human IVD is an immune-privileged organ with functional FasL expression. Studies have shown that the Fas-FasL apoptosis mechanism maintains immune privilege by interacting with invasive immune cells 36, 54.

IDD is characterized by FasL dysfunction and an imbalance in the interaction between NPCs and immune cells 55. In the presence of HNPCs with high FasL expression, macrophage apoptosis and CD8+ T cells were significantly increased 44. In addition, FasL overexpression in NPCs can also induce cytotoxic T lymphocyte apoptosis via invasive Fas-positive cells, thus contributing to NP immune privilege 56. Increasing evidence shows that FasL maintains IVD immune privilege by inducing immune cell apoptosis 57, 58 (Table 1).

Previous studies have shown that sFas and sFasL are closely associated with the immune mechanism of IVD (Table 1). sFas is produced by proteolysis of membrane-bound receptors or mRNA splicing. Due to the lack of a transmembrane domain, sFas has a protective effect on Fas-mediated apoptosis 59. sFas is considered a marker of many autoimmune diseases and tumors because sFas can promote proliferation by inhibiting immune or tumor cell apoptosis 60-62. A decrease in sFasL may protect infiltrating immune cells from apoptosis and exacerbate NPC loss 63. In the NP, an increase in sFas can neutralize the effect of FasL, reduce the ability of sFasL to induce infiltrating immune cell apoptosis, promote the infiltration of immune cells, and promote IDD occurrence by breaking the NP immune balance. Fas-mediated apoptosis is also regulated by immune factors and signaling pathways, such as T cells, B cells, and apoptotic and antiapoptotic signaling pathways 64.

Mesenchymal stem cell (MSC) transplantation can regenerate degenerated IVDs to support health and enhance immunity 55. However, there are no apparent advantages or disadvantages regarding the immune response in IDD 65. The immune response has a protective effect under normal conditions. When IDD occurs, the NP and other factors are exposed to the immune system, and the immune response causes further damage, which may be a warning signal. However, the primary mechanism of IDD-associated immunity and Fas-mediated apoptosis has not been fully elucidated. Further understanding the mechanism of immune privilege may provide a new target to cure IDD.

### Participation in angiogenesis

The IVD is the largest nonvascular organ in the human body. With the development of IDD, the IVD is gradually vascularized 66. Increasing evidence shows that IDD has multiple angiogenesis mechanisms 67. Abnormal changes in the ECM induce inward vascular growth 68. Degenerated NP tissue interacts with vascular endothelial cells (VECs) to stimulate these cells to grow inward. The traditional IVD structure is a crucial factor in promoting its avascular state. However, whether there is an effective mechanism to prevent IVD vascularization is still unknown.

It is well known that both HNPCs and VECs express Fas/FasL, and IDD angiogenesis is associated with Fas/FasL (Table 1). Studies have shown that the regulation of endothelial Fas signaling promotes vascular integrity by mural cells in tumors 69. Fas-mediated apoptosis plays an essential role in preventing angiogenesis in other tissues 70, 71. The ability of normal NPCs to induce VEC apoptosis is stronger than that of degenerated NPCs and is mediated by Fas/FasL 72. FasL expression is highly developed in the early stage of IVD vessel degeneration 73, 74. However, with IVD worsening, FasL expression decreases during angiogenesis and other pathological processes 75. FasL mediates downstream FADD and caspase-3 and induces VEC apoptosis, thus preventing IVD angiogenesis 72. VEC apoptosis can lead to decreased microvessel density in the endplate and affect the pathological process of IDD 76. Therefore, HNPCs can induce VEC apoptosis and inhibit VEC migration. However, with increasing age, NPCs lose the ability to induce apoptosis.

The changes in the IVD microenvironment may be a strong inducer of abnormal vascular growth in IDD. The FasL-Fas network plays a vital role in the molecular mechanism of inhibiting angiogenesis in the human IVD through interactions with VECs 77. The normal NP avascular state is due to a simple physiological barrier and the Fas-FasL network as an active molecular mechanism. In addition to the traditional barrier, FasL may be a molecular monitor associated with avascular maintenance of the IVD by inducing VEC apoptosis. FasL can increase Fas expression in VECs, enhancing the effect of the FasL-Fas interaction on apoptosis 40. Therefore, FasL plays an intricate role in IVD vascularization. Scholars have identified the inducing factor of IDD. The Fas pathway also inhibits IVD vascularization in late-stage IDD. This finding may suggest that vascularization of the IVD is not necessarily a harmful factor for IVD.

### Inhibiting Fas pathway to slow IDD

#### RNA interference

In recent years, IDD treatment at the RNA level has become a hot topic. Increasing evidence shows that apoptosis is regulated by microRNAs (miRNAs), and inhibiting the death receptor pathway by RNA targeting can effectively slow IDD (Table 2). Wang et al. 56 used lentivirus carrying the miR-155 precursor (pre-miR-155) to transfect human NPCs. After transfection, the expression of FADD and caspase-3 in NPCs was inhibited. After miR-155 was knocked out, FADD and caspase-3 were overexpressed. Uncontrolled miR-155 promoted IVD cell apoptosis by targeting FADD and caspase-3, indicating that miR-155 is a potential suppressor gene of the Fas pathway. In addition, Zhang et al. 78 found that the downregulation of miR-210 could promote HNPC apoptosis mediated by the death receptor. The proportion of apoptotic NPCs decreased significantly after pre-miR-210 treatment. miR-210 may also be a new target for IDD treatment. Similarly, Li et al. 79 showed that miR-129-5p had a protective effect against IDD. The upregulation of miR-129-5p can promote the proliferation of NPCs and reduce the expression of FADD in IDD rats. In addition to that in NPCs, miR-34a was significantly increased in human degenerative CEP cells, accompanied by increased apoptosis mediated by the death receptor pathway 80. Bcl-2 is the target of miR-34a, and miR-34a can also promote apoptosis by inhibiting Bcl-2, which is achieved through the mitochondrial pathway 80. Silencing miR-34a may be a new intervention strategy for IDD treatment by preventing CEP cell apoptosis.

Long noncoding RNAs (lncRNAs) are a group of nonprotein-coding RNAs with key functions in various human diseases 81. In IDD tissues, the expression of lncRNA MAGI2-AS3 was downregulated. Cui et al. 82 collected the serum of 66 IDD patients and 58 healthy volunteers. Real-time quantitative PCR (qRT-PCR) and Western blot analysis showed that lncRNA MAGI2-AS3 could inhibit FasL expression in NPCs, which may be a potential therapeutic strategy. However, the role of FasL in IVD has not been fully elucidated. Further examination is needed to inhibit IDD by upregulating FasL.

It has been reported that Fas small interfering RNA (siRNA) has a better effect on IVD cell apoptosis (approximately 10%) than platelet-derived growth factor (PDGF) and insulin-like growth factor-1 (IGF-1) (2% and 5%, respectively) 83. siRNA-mediated Fas inhibition can increase cell proliferation by approximately 21%. In the early stage of apoptosis, Fas downregulation can reduce apoptosis and induce proliferation, resulting in enhanced IVD cell viability. Fas siRNA may be an effective method for treating IDD 84. In addition, it has been reported that RNA interference is a promising method of local IDD therapy 85. In conclusion, it is theoretically feasible to slow IDD by inhibiting the Fas pathway at the RNA level (Table 2). However, it is still unknown whether it is possible to inhibit Fas expression at the RNA level due to the complex regulatory mechanisms of IDD and the mutual restriction of various factors.

### Cell secretory factors

Cytokines are a class of proteins with extensive biological activities that regulate cell growth, cell differentiation, and various biological effects by binding to corresponding receptors. Transforming growth factor-β1 (TGF-β1) has the potential to regenerate the IVD. Xie et al. 86 showed that TGF-β1 significantly reduced apoptosis in rat NPCs treated with TNF-α and decreased the activities of caspase-3 and caspase-8.

With increasing age, notochord cells (IVD precursor cells) in the NP gradually disappeared. Erwin et al. 87 found that notochord cell secretory factors can inhibit NPC death by inhibiting activated caspase-9, caspase-3, and caspase-7, upregulating genes (aggrecan, collagen type 2, CD44, link protein, and TIMP-1), and promoting anabolic activity and NP protection and protect NP tissue by promoting ECM synthesis. Utilizing the resilience of notochord cells may lead to new cellular and molecular strategies in IDD therapy.

Islet amyloid polypeptide (IAPP) is involved in the regulation of glucose metabolism and apoptosis. During IDD, the expression of IAPP in AFCs decreases significantly 88. The downregulation of IAPP induces reactive oxygen species production, decreases MMP, and increases Ca^2+^ concentrations in AFCs, eventually leading to cell death. The downregulation of IAPP promotes cytochrome C release in mitochondria, resulting in the activation of caspase-3 and caspase-9 and the Fas/FasL system and cell death. In conclusion, the downregulation of IAPP induces human AFC death, which may provide a new therapeutic target for IDD treatment 88.

Lysyl oxidase (LOX) can protect chondrocytes from TNF-α-induced apoptosis and plays an antiapoptotic role in TNF-α-treated rat NPCs, suggesting that this factor may be a promising agent in the treatment of IDD. The expression of LOX was significantly decreased in NPCs treated with TNF-α. Exogenous LOX can maintain TNF-α-induced NPC activity, reduce the apoptosis rate, and promote the secretion of ECM 89. Further molecular mechanism studies showed that LOX inhibited the Fas/FasL and p53 pathways 89. Therefore, TGF-β1, notochord cell secretory factors, IAPP, and LOX may become effective treatment strategies, but clinical trials are needed to verify their effectiveness and feasibility (Table 2).

In addition to the above methods, other treatment studies have also been performed. Paeoniflorin has a protective effect on cells. It has been reported that paeoniflorin can inhibit activation of the IVD death receptor pathway and reduce AFC apoptosis 37. In addition, MSCs express FasL at the gene level but not the protein level. MSC transplantation may maintain the immune privilege of the IVD by differentiating transplanted MSCs into FasL-expressing cells, thus effectively promoting degenerative IVD regeneration 90. In conclusion, Fas pathway inhibition may be an effective way to treat IDD. It is necessary to further study the mechanism of the death receptor pathway in IDD.

## The mitochondrial pathway and IDD

### The mitochondrial pathway induces IDD

Mitophagy can lead to cell death by eliminating abnormal or damaged mitochondria and controlling the quality and quantity of mitochondria to maintain dynamic balance and resist oxidative stress. Oxidative stress rapidly promotes mitochondrial movement through c-Jun N-terminal kinase (JNK) in the early stage but decreases mitochondria and increases apoptosis in the late stage 91. In IDD, Bax is upregulated, and Bcl-2 is downregulated. Bax leads to mitochondrial rupture by opening voltage-dependent anion channels in mitochondria, while Bcl-2 protects normal IVD tissue from apoptosis 7. Bcl-2 binds to Bax and inhibits the proapoptotic function of Bax. Changes in the balance between Bcl-2 and Bax lead to a proapoptotic signal 92.

The mitochondrial pathway is involved in inflammation-related IDD. IL-1β is highly expressed in degenerative IVDs. Shen et al. 93 showed that IL-1β promotes NPC apoptosis in the human degenerative IVD and is associated with mitochondrial pathway activation. IL-1β reduces the Bcl-2/Bax ratio, enhances cytochrome C release by mitochondria into the cytoplasm, and induces mitochondrial-mediated apoptosis. Subsequently, mitochondria are damaged under IL-1β stimulation and help activate autophagy. Simultaneously, autophagy caused by damaged mitochondria has a protective effect on cell apoptosis, suggesting a positive feedback mechanism under inflammatory stress conditions 93. Eser et al. 94 found a specific relationship between the Bcl-2 GG genotype and IDD, and the proportion of Bax-positive cells was associated with the IDD grade. Shang et al. 95 also reported a relationship between the Bcl-2 gene polymorphism and IDD occurrence and severity. The researchers found that the - 938C > A polymorphism of the Bcl-2 gene was associated with IDD occurrence and severity in the Chinese Han population. In addition, Dagistan et al. 96 reported that Bax might be a key pathophysiological factor in patients with symptomatic IDD.

Under serum deprivation conditions, caspase-9, caspase-3, PARP, cytochrome c, and Bax were are highly expressed, and serum deprivation induced apoptosis occurred in rat CEP cells through the mitochondrial apoptotic pathway 97. Diabetes is an essential factor in IDD 98. The accumulation of advanced glycation end products (AGEs) is a necessary factor in diabetic IDD. AGEs induce rabbit AFC apoptosis, which is closely associated with activation of the mitochondrial apoptosis pathway 99. In summary, the mitochondrial apoptosis pathway is an important cause of IDD (Table 3).

### Inhibiting the mitochondrial pathway to slow IDD

#### MSC transplantation

BMSC coculture can increase NPC viability and reduce NPC apoptosis. Furthermore, BMSCs can alleviate compression-induced mitochondrial damage by lowering reactive oxygen species levels and maintaining mitochondrial membrane potential and mitochondrial integrity. In addition, coculture with BMSCs inhibited the activation of caspase-3 and caspase-9, decreased cytochrome c and Bax expression, and increased Bcl-2 expression 100. Moreover, AFC apoptosis induced by IL-1β was attenuated by inhibiting mitochondrial function after coculture with BMSCs. The paracrine effect of BMSCs has anti-inflammatory and antiapoptotic effects on IDD and is at least partially mediated by the mitochondrial apoptotic pathway in AFCs 101. These studies enhance the understanding of MSC-based IDD treatment.

### Inhibiting oxidative stress

Oxidative stress is a fundamental cause of IDD. Oxidative stress induces mitochondrial apoptosis in NPCs 102. Inhibiting oxidative stress can protect NPCs from apoptosis. MitoQ, a mitochondria-targeted antioxidant, eliminates damaged mitochondria, improves redox balance, and increases cell survival. Compression can also promote oxidative stress, mitochondrial dysfunction, and NPC apoptosis, and MitoQ can reduce IDD in isolated rat compression models 103. MitoQ prevents IDD by improving mitochondrial dysfunction and redox imbalance. Advanced oxidation protein products (AOPPs) are new biomarkers of oxidative stress. Higher levels of AOPPs have been found in human IDD. Allicin can protect NPCs from AOPP-mediated oxidative stress and mitochondrial dysfunction by inhibiting the p38 MAPK pathway 104. Allicin may be a promising treatment for AOPP-mediated oxidative stress in the progression of IDD.

After stimulation by AGEs, the mitochondrial apoptotic pathway is activated, and the accumulation of AGEs in NP tissue parallels IDD progression, forming an oxidative stress microenvironment 105. AGEs can significantly increase the production of mitochondrial reactive oxygen species, prolong the activation of the mitochondrial permeability transition pore, increase the protein level of mitochondrial Bax and decrease the protein level of Bcl-2 105. Yu et al. 105 found that the impaired function of sirtuin3 (SIRT3) and the mitochondrial antioxidant network are essential mechanisms by which AGEs induce oxidative stress and secondary human NPC apoptosis. SIRT3 can prevent AGE-induced human NPC apoptosis and IDD. Targeting SIRT3 to improve mitochondrial redox homeostasis may be a potential therapeutic strategy to reduce IDD associated with AGEs.

H_2_O_2_ significantly impairs AFC activity in a dose- and time-dependent manner and induced AFC apoptosis through the mitochondrial pathway 106. TGF-β1 can downregulate the expression of mitochondrial-related apoptotic proteins (Bax/Bcl-2, caspase-9) and downregulate H_2_O_2_ by upregulating the expression of glutathione peroxidase-1 (GPx-1). TGF-β1 attenuates autophagy and apoptosis induced by exogenous H_2_O_2_ by downregulating extracellular signal-regulated kinases (ERKs) in AFCs 106.

### Cell secretory factors

NPC apoptosis exacerbates IDD, and inhibiting NPC apoptosis through the mitochondrial pathway can improve IDD. Cortistatin (CST) expression in NPCs is decreased during senescence and TNF-α-induced IDD. Exogenous CST treatment can reduce TNF-α-mediated catabolism and apoptosis 107. Additionally, CST can inhibit mitochondrial dysfunction in NPCs and prevent IDD by targeting mitochondrial reactive oxygen species (ROS)-dependent NLRP3 inflammasome activation 107.

Heme oxygenase-1 (HO-1) is downregulated in IDD tissues, which reduces the senescence of NPCs by inducing autophagy via the mitochondrial pathway. HO-1 overexpression can reduce NPC aging, protect mitochondrial function, and promote NPC autophagy through the mitochondrial pathway. *In vivo*, HO-1 overexpression inhibits IDD and enhances autophagy 108. Under nutrient deficiency conditions, the mitochondrial apoptosis pathway is activated, and NPCs exhibit decreased antiapoptotic Bcl-2 and increased Bax, caspase-3, and caspase-9 expression. RhSIRT1 can inhibit these changes, and autophagy induced by rhSIRT1 can inhibit cell apoptosis 109.

NPCs were pretreated with pyrroloquinoline quinone (PQQ) and showed increased cell viability, which helped to maintain mitochondrial membrane potential and reduce apoptosis. In the presence of PQQ, cells could support the production of ECM even in an apoptotic environment. In addition, PQQ treatment increased the expression of Bcl-2, inhibited cytochrome c release, and reduced Bax and caspase-3 expression. PQQ protected rat NPCs from oxidative stress through the mitochondrial-mediated pathway 110. These findings suggested that PQQ could be used as a potential drug to prevent IDD.

These studies provide understanding of the therapeutic targets of IDD (Table 4). The intrinsic mitochondrial apoptosis pathway represents a promising target for new therapies. Successful targeting of this pathway has the potential to treat a variety of diseases 111. Mitochondria are also potential therapeutic targets for IDD 112. Inhibition of the intrinsic caspase-9 pathway significantly inhibited serum deprivation-induced apoptosis. Although caspase-8 inhibitors cannot reduce apoptosis, caspase-9 inhibitors can be used as potential treatments for IDD 97. Caspase is the executor of apoptosis in both the death receptor and mitochondrial pathways. Caspase inhibitors may be used as antiapoptotic agents. With further basic research, some drugs have been gradually used in clinical treatments. Palmlintide is an approved islet amyloid peptide analog that is responsible for regulating energy homeostasis and may play a key role in reversing IDD. First, this drug can promote NPC survival through mitochondrial-mediated Bcl-2/caspase-3-dependent apoptosis. Second, it can improve NPC proliferation, glycolytic activity, and ECM synthesis 113. These findings provide a new potential pharmacological treatment strategy.

### ERS and IDD

#### ERS induces IDD

The ER is the hub between environmental signals and cell biological functions, and abnormal ER function can lead to various diseases. ERS and mitochondria participate in IVD cell apoptosis 114. In addition to the mitochondrial pathway, ERS can also induce apoptosis in rat AFCs by cyclic stretching 115.

Low pH induces apoptosis in rat NPCs. Long-term exposure to acidic substances can reduce cell viability and upregulate ERS markers 116. ERS is involved in acid-induced NPC apoptosis. Studies have shown that acid-sensing channel 1a (ASIC1a) partially regulates ERS and promotes NPC apoptosis in response to acid stimulation and may be a new therapeutic target for IDD 117. ERS is involved in the natural degradation of the NP, and it is also key to NPC acid tolerance and the alleviation of acid-induced NPC apoptosis. Acid induces ERS in NPCs at the gene and protein levels. However, this response does not precisely match and may involve translation and posttranslational modifications. In the early stage, ERS and the subsequent UPR were activated, which increased the NPC survival rate. This protective mechanism needs further study and may involve autophagy and cell quiescence associated with the ER 116.

### Inhibiting the ERS pathway

NPC apoptosis is associated with ERS in the IVD. In degenerated IVD tissues, ERS levels and apoptosis rates are increased. ERS can promote NPC apoptosis and IDD. Since ERS is a newly discovered apoptosis pathway, few targeted studies on ERS inhibition have been performed. Under hypoxic conditions, NPCs show a reduced ERS response and hypoxia, and HIF-1α attenuates the ERS response in NPCs 118. H2S has attracted much attention because of its antiapoptotic effect on degenerative diseases. The protective mechanism of H2S in IDD involves the inhibition of ER and mitochondrial dysfunction in NPCs 119. Liao et al. 120 found that MSC-Exos can reduce ERS-induced apoptosis by activating Akt and ERK signaling. This study highlights the therapeutic role of exosomes in preventing the progression of IDD.

## Research limitations

These studies show that it is feasible to treat IDD by inhibiting apoptosis and provide ideas for targeted therapy. However, several difficulties still need to be resolved. First, the etiology and mechanism of IDD have not been fully elucidated, and IDD treatment lacks specificity. Second, comparable changes in apoptosis and proliferation were not observed in the above experiments. Cell metabolic activity and the ability to produce ECM are still critical indicators of IVD repair, and experiments to address these factors have not been performed. In addition, the construction of a targeted therapeutic vector is difficult. Finally, most of the treatments have been carried out in animal models, and there is a lack of sufficient clinical evidence to prove the feasibility and effectiveness of their clinical application.

## Summary

In conclusion, the death receptor pathway, mitochondrial pathway, and ERS pathway are involved in IDD occurrence and development. Targeted therapy for IDD is the future, and the apoptosis pathway may be an important targeted therapy. Although previous studies have some limitations, the current research results have shown the great potential of drug-targeted inhibition of the apoptosis pathway to cure IDD.

## Figures and Tables

**Table 1:** Summary of Fas pathway promotion of IDD

Research type	Molecule	Influence	Reference
Human serum	FasL-844 TT and FasL-844 T/C	Associated with more severe IDD and waist burden, smoking, and age.	36
Human serum	Fas-1377 G/A and FasL-844 C/T	Associated with IDD severity in the Chinese Han population.	43
Human serum	Fas G allele (rs1800682)/FasL T allele (rs763110)	Increased IDD risk/decreased IDD risk.	45
HNPCs	IL-2	Inhibit cell proliferation and induce Fas pathway and ECM degradation.	49
HNPCs	Fas/FasL	Produce IDD-related pro-inflammatory cytokines.	50, 51
Human immune cells	Fas-FasL	Promote immune cell death and maintain immune privilege.	36, 44, 54, 56-58
Human immune cells or tumor cells	sFas	Inhibit cell apoptosis and promote proliferation.	60-62
HNPCs	sFasL	Induce infiltrating immune cells apoptosis and reduce the loss of NPCs.	63
Human VECs	Fas/FasL	Induce cell apoptosis.	40, 70-72, 77

**Table 2:** Summary of possible targets of Fas pathway inhibition

Research type	Therapeutic molecules	Influence	Reference
HNPCs/Rat NPCs	pre-miR-155, miR-210/miR-129-5p	Inhibit Fas apoptosis pathway and promote NPC proliferation	56, 78, 79
Human CEP cells	miR-34a	Activate the death receptor pathway and inhibit Bcl-2.	80
Plasma of IDD patients	LncRNA MAGI2-AS3	Participate in the regulation of FasL expression in NPCs.	82
Rat disc cells	Fas siRNA	Induce cell proliferation and improve the viability of IVD cells.	83, 84
Rat NPCs	TGF-β1	Significantly reduce NPCs apoptosis induced by TNF-α and decrease caspase-3 and caspase-8 activities.	86
Bovine NPCs	Notochord cell secretory factors	Inhibit NPCs death and promote ECM synthesis.	82
Human AFCs	IAPP	Inhibit the Fas pathway and cytochrome c release.	88
Rat NPCs	LOX	Reduce the rate of apoptosis and improve ECM secretion in TNF-α-treated cells. Inhibit the Fas/FasL and p53 pathways.	89
Rat AFCs	Paeoniflorin	Inhibit activation of the IVD death receptor pathway.	37
Canine IVD	MSCs	Differentiate into FasL-expressing cells to maintain IVD immune privilege and promote IVD regeneration.	90

**Table 3:** Summary of mitochondrial pathway promotion of IDD

Research type	Molecule	Influence	Reference
Human NPCs	IL-1β	Reduce the Bcl-2/Bax ratio, enhance cytochrome C release into the cytoplasm and induce mitochondrial-mediated apoptosis.	93
Gene polymorphism	Bcl-2	Related to the occurrence and severity of IDD.	94, 95
Rat CEP cells	Serum deprivation	Activate the mitochondrial apoptosis pathway.	97
Rabbit AFCs	Accumulation of AGEs	Activate the mitochondrial apoptosis pathway.	99

**Table 4:** Summary of possible targets of mitochondrial pathway inhibition

Research type	Therapeutic molecules	Influence	Reference
Rat NPCs/AFCs	BMSCs	Lower the reactive oxygen species level and maintain mitochondrial membrane potential and mitochondrial integrity.	100, 101
Rats NPCs	MitoQ	Improve mitochondrial dysfunction and redox imbalance.	103
HNPCs	Allicin	Protect NPCs from AOPP-mediated oxidative stress and mitochondrial dysfunction by inhibiting the p38 MAPK pathway.	104
HNPCs	SIRT3	Prevent HNPC apoptosis and IDD caused by AGEs.	105
Rat AFCs	TGF-β1	Attenuate autophagy and apoptosis induced by exogenous H2O2.	106
Human NP tissue and mouse NPCs	CST	Reduce TNF-α-mediated catabolism and apoptosis. Inhibit mitochondrial dysfunction and prevent IDD by targeting mitochondrial ROS-dependent NLRP3 inflammasome activation.	107
HNPCs	HO-1/RhSIRT1	Protect mitochondrial function and promote NPC autophagy.	108, 109
HNPCs	PQQ	Increase cell viability, maintain mitochondrial membrane potential, and inhibit oxidative stress	110
HNPCs	Palmlintide	Promote the survival of NPCs and improve NPC proliferation, glycolytic activity, and ECM synthesis.	113

## Abbreviations

IVD: intervertebral disc
IDD: intervertebral disc degeneration
LBP: low back pain
ECM: extracellular matrix
ER: endoplasmic reticulum
ERS: endoplasmic reticulum stress
NP: nucleus pulposus
NPC: nucleus pulposus cell
TNF-α: tumor necrosis factor-alpha
IL: interleukin
MMP: matrix metalloproteinase
AFCs: annulus fibrosus cells
CEP: cartilaginous endplate
PCD: programmed cell death
TRAIL: TNF-related apoptosis-inducing ligand
FADD: Fas-associated protein with the death domain
DISC: death-inducing signaling complex
NIK: NF-κB-induced kinase
IKK: IκB kinase
Bcl-2: B-cell lymphoma-2
IAPs: inhibitor of apoptosis proteins
Smac: second mitochondria-derived activator of caspase
DIABLO: direct inhibitor of apoptosis-binding protein with low pl
UPR: unfolded protein response
RNA: ribonucleic acid
PERK: endoplasmic reticulum kinase
IRE1: inositol-requiring enzyme 1
ATF6: transcription factor 6
eIF-2α: eukaryotic translation initiation factor 2α
ATF4: transcription factor 4
CHOP: C/EBP-homologous protein
XBP1: X box binding protein 1
mRNA: messenger RNA
HNPCs: human nucleus pulposus cells
MSCs: mesenchymal stem cells
VECs: vascular endothelial cells
miRNA: microRNA
lncRNA: long noncoding RNA
qRT-PCR: Real-time quantitative PCR
siRNA: small interfering RNA
PDGF: platelet-derived growth factor
IGF-1: growth factor-1
TGF - β1: transforming growth factor - β1
IAPP: Islet amyloid polypeptide
LOX: lysyl oxidase
JNK: c-Jun N-terminal kinase
AGEs: advanced glycation end products
AOPP: advanced oxidation protein products
SIRT3: sirtuin3
GPx-1: glutathione peroxidase-1
ERK: extracellular signal-regulated kinases
CST: cortistatin
ROS: reactive oxygen species
HO-1: heme oxygenase-1
PQQ: pyrroloquinoline quinone
ASIC1a: acid-sensing channel 1a
